# Novel and classic approaches for managing gastrointestinal cancers

**DOI:** 10.1002/ags3.12670

**Published:** 2023-03-28

**Authors:** Koshi Mimori

**Affiliations:** ^1^ Kyushu University Beppu Hospital Beppu Japan

This issue comprises 12 original articles and three reviews. The original articles focus on topics such as use of multiple chemotherapies, novel surgical techniques, reconsideration of current approaches, and comorbidities and postoperative outcomes. Three reviews summarize immune checkpoint inhibitor (ICI) treatments for colorectal cancer, various aspects of rectal cancer, and pathological consideration of tumor deposits.

Because immunotherapy has been established as an additional treatment approach, multimodal therapy has become mainstream for treating gastrointestinal cancers. This issue includes four original articles on “novel multimodal treatment approaches.” Kurokawa et al.^1^ have introduced a phase II clinical trial of chemotherapy for gastric and esophagogastric junction (EGJ) adenocarcinoma. They found that neoadjuvant docetaxel, oxaplatin, and S‐1 chemotherapy showed favorable antitumor effect and tolerable safety profile in patients with gastric or EGJ adenocarcinoma (DOI: 10.1002/ags3.12632). Matsumura et al. have demonstrated that neoadjuvant chemoradiotherapy with S1 (S1‐NACRT) was effective for resectable pancreatic duct adenocarcinoma.^1^ This regimen provided tolerability, favorable local control, and comparable survival benefits (DOI: 10.1002/ags3.12624). Kuwahara et al. have described that advances in medical treatment, such as antitumor necrosis factor‐alpha agents for patients with ulcerative colitis, have improved the rate of remission induction and increased the number of patients for whom surgery can be avoided. However, as these patients have a longer disease duration, the number of patients with cancer and dysplasia owing to inflammation is increasing. Therefore, the authors caution that there is a need for care when malignancies are treated with biological agents, especially in older patients (DOI: 10.1002/ags3.12626). Yin et al. have reported the liver transplantation (LT) rate after transarterial chemoembolization (TACE) for hepatocellular carcinoma cases. LT significantly improved the overall survival rate; however, the LT rate decreased if patients underwent more than three TACE procedures (DOI: 10.1002/ags3.12622).

The following three papers introduce “innovation in surgical procedures.” Ito et al. have presented a novel technique to prevent postoperative pancreatic fistulas after pancreas‐preserving duodenectomy.^2^ The technique involved identifying the accessory pancreatic duct using indocyanine green fluorescent imaging followed by closing the duct (DOI: 10.1002/ags3.12619). Zhang et al. have reported about liver resection by considering the anatomy of Laennec's capsule, updating ideas about liver anatomy, and providing a new approach for normalization and standardization of liver surgery methods (DOI: 10.1002/ags3.12618). Tominaga et al. have reported that sigmoid colon cancer patients with body mass index ≥25 kg/m^2^ are the most appropriate candidates for postoperative transanal drainage tube (TDT) insertion. They found that in patients with rectal cancer, TDT use can prevent anal leakage (DOI: 10.1002/ags3.12634).

Articles in this issue also address “reconsidering the unchangeable, immutable approaches” in GI care. For example, papers consider novel perspectives of anatomy for operative safety, optimal timing for treatment of metastatic sites, and use of conventional serum tumor markers to measure malignant potential. Watanabe et al. have reported the importance of lymph node dissection to detect metastases in splenic flexure colon cancer cases.^3^ Given the frequency of lymph node metastasis, the authors concluded that the accessory middle colic artery should be targeted for dissection (DOI: 10.1002/ags3.12620). Kasai et al. have reported the long‐term outcomes of staged liver resection for synchronous liver metastases (SLM) from colorectal cancer (CRC). Patients diagnosed with resectable SLM from CRC underwent resection of CRC followed by resection of SLM 2–3 months later without preoperative chemotherapy. They reported that non‐early recurrence cases had significantly better overall survival (OS) rates than cases with early recurrence (ER) or unresectable recurrence (UR). There was no significant difference in the OS rate between the ER and UR groups. Therefore, the malignant potential of the ER CRC cases was almost equal to those of the UR CRC cases. The authors disclosed the increase in serum CEA before hepatectomy was a risk factor for early recurrence, and could be useful when considering the treatment strategy for SLM from CRC. They proposed that an optimal treatment strategy for SLM from CRC should be established, including the timing of liver resection and the combination of chemotherapy, based on prognostic factors such as the conventional serum CEA level (DOI: 10.1002/ags3.12628). Takagi et al. have reported that the combined evaluation of three tumor markers, namely carbohydrate antigen 19–9 (CA 19–9), carcinoembryonic antigen (CEA), and duke pancreatic monoclonal antigen type 2 (DUPAN‐2), may provide helpful information for the management of patients with pancreatic ductal adenocarcinoma (DOI: 10.1002/ags3.12629).

Two papers elaborate on “preexisting conditions and postoperative complications,” which have been long‐standing issues in the management of gastrointestinal cancers. Sakai et al. have stated that tracheobronchial necrosis (TBN) is a severe and potentially life‐threatening complication after esophagectomy for laryngeal, pharyngeal, and esophageal cancers.^4^ However, analysis of clinical data of 6370 patients who underwent esophagectomy showed that the incidence of primary TBN was lower than expected. Preventing worsening TBN requires the maintenance of tracheal blood flow (DOI: 10.1002/ags3.12625). Fukushima et al. have reported the clinical outcome after gastrectomy for gastric cancer in patients who had preoperative osteopenia. Systemic low bone mineral density was an independent and distinctive risk factor for poor OS and disease‐free survival (DOI: 10.1002/ags3.12635).

Finally, this issue includes three particularly outstanding and informative review papers. All these papers have been well‐researched and have summarized the most critical problems at present. Takemasa et al.^5^ have outlined various types of precision medicine approaches to care for rectal cancer. They reviewed studies cover minimally invasive surgery for rectal cancer; prediction of surgical complications; visualization of procedures; multimodal therapies; molecular technologies for cancer treatment, including liquid biopsy; diagnosis of rectal cancer; and complications after anterior resection, also known as low anterior resection syndrome (DOI: 10.1002/ags3.12646). This Associate Editor confidently recommends the current review as it comprehensively covers recent topics concerning diverse aspects of rectal cancer from multiple perspectives. Miyamoto et al. have provided a relevant and comprehensive summary of clinical studies of ICI treatment for CRC cases with high microsatellite instability. This paper referred to the gut microbiota and the efficacy of ICIs combined with VEGF inhibitors as future perspectives (DOI: 10.1002/ags3.12633). Finally, Ueno et al. have described tumor deposit (TD) categorization in the TNM classification. They concluded that the number of TDs in tumor staging should be determined to optimize the prognostic value of TNM classification and reproducibility. Consideration of TDs should help determine the appropriate TNM staging and benefit of CRC cases by optimizing treatment decisions (DOI: 10.1002/ags3.12652).

## CONFLICT OF INTEREST STATEMENT

No conflict of interests.
